# Emergence and evolution of standardization systems in medical biology laboratories

**DOI:** 10.1515/almed-2024-0014

**Published:** 2024-05-01

**Authors:** Rita B. Khattar, Maha E. Nehme

**Affiliations:** 507466 Holy Family University, Batroun, Liban-Nord, Lebanon

**Keywords:** standardization, medical laboratories, ISO

## Abstract

**Introduction:**

The history of standardization relating to the activities of medical laboratories traces the development of quality system standards in the world, and their evolution.

**Content:**

In this study, we have included the key benchmarks that represent the stages of the quality system’s evolution in recent decades. Accreditation of medical laboratories has become compulsory in most countries, regarding national or international standards. International acknowledgment of the effectiveness of the results delivered to the many stakeholders, particularly patients and prescribers, is conferred through the use of standards.

**Summary:**

The ISO 15189 standard represents the latest and most specific international standards for medical laboratories of all types.

**Outlook:**

More research is necessary to study if laboratory practices reflect the evolution of standards within the medical laboratory field.

## Introduction

A medical laboratory’s goal is to guide for a medical diagnosis, which may be delayed by inaccurate results, insufficient therapies, and unnecessary test requests; as a result, this can lead to an increase in costs, time loss, and over-investment in human resources [1].

Worldwide, laboratories have recognized how essential it is for them to operate within a highly effective global quality management system that ensures the best execution of all processes in order to correct errors emerging from these processes and their consequences [2, 3]. Standardized repositories were then developed, enabling the use of techniques for identifying non-conformities at each stage of the analysis. However, putting in place a strong quality management system, “allows for a high-quality laboratory capable of detecting errors and preventing their recurrence, but does not guarantee an error-free laboratory” [1].

Gradually, the benefits of establishing an effective “quality system” became the focus of health professionals. These benefits included increased traceability and quality of results [2, 3], decreased laboratory errors [1], decreased costs associated with poor quality [4, 5], standardization of techniques, and cooperation among all team members for the implementation of the quality system.

Therefore, effective standardization promotes team members’ perceptions of their values, good internal communication, and employee empowerment [2, 6].

The goal of this review is to give a general overview of the development and evolution of the standards used by quality management systems in medical laboratories.

## Standardization: definition and typology

“A standard allows for the establishment of guidelines and a procedure to be followed for operations related to all different sectors.” It determines the optimal process to adopt during the activity of a company in order to combine efficiency, safety, and reliability [7].

Standardization may be done on an international, national, or local level. The ISO is the most significant of the global standardization bodies. The National Committee for Clinical Laboratory Standards (NCCLS), currently known as the Clinical and Laboratory Standards Institute (CLSI), develops standards and policies that are helpful to the medical field. Additional examples are the WHO and the CEN (European Committee for Standardization).

International standards, however, might be challenging to adapt to a given country given their lack of specificity. National standards can be developed based on international standards and customized to the requirements of the country in question [1].

The American National Standards Institute (ANSI), the Standardization Administration of China (SAC), the Deutsches Institut für Normung (DIN), the British Standards Institution (BSI), the Japanese Industrial Standards Committee (JISC), and the Canadian Standards Association (CSA) are various nations’ equivalents of the French agency for Standardization AFNOR which officially consult each draft French, European, or international standard in France [8].

## Emergence and evolution of standardization in medical laboratories

The United States was the first to take an interest in the quality in medical analysis laboratories. As early as the 1960s, the CLIA (Clinical Laboratory Improvement Act) (81 Stat. 536, Public Law 90–174, Sec. 5) was drafted in 1967 to establish quality standards for laboratory tests performed on human samples [9].

Additionally, NCCLS formally established in 1968, released in 1969 “Preparation of Manuals for Installation, Operation and Repair of Laboratory Instruments,” a guideline for quality standards. 20 laboratory standards were established during the following ten years. The NCCLS started to gain recognition on a global scale in 1985, and the WHO named it as one of its Collaborating Centers for Medical Laboratory Standards. In addition, to cover all laboratory domains, the three basic NCCLS committees (clinical chemistry, hematology, and microbiology) have been expanded to eight [10].

In 1988, CLIA’88 was declared to replace CLIA’67 by the Public Health Services Act Amendment (102 Stat. 2903, Public Law 100-578) [9]. Implemented in February 1992 [11], its goal was to improve all medical testing laboratories in the United States and ensure the quality of their tests, wherever they were performed [1], and this program is still used nowadays under the same name CLIA’88 despite two subsequent revisions, one in 1997 and another in 2012 [9, 11].

In fact, the College of American Pathologists (CAP) uses CLIA as part of the Laboratory Accreditation Program (LAP) for the accreditation of clinical laboratories all over the world, giving it an international aspect even though it is a very specific national regulation in the United States to support quality practices. Except for clinical trials and basic research, it is a document that applies to all clinical laboratory tests performed on humans covering specific disciplines such as for histopathology or genetic testing.

CLIA is a program focused primarily on process management and control, much more than on customers and staff [12]. It contains several sub-parts dealing with, among other things, the general provisions (Subpart A), the standards and conditions for proficiency testing by specialty and sub-specialty (Subpart H), as well as the general quality system of the laboratory and the 3 pre-post-analytical phases, also detailing it by specialty and sub-specialty (Subpart K) [13].

The established guidelines and rules have improved the quality of analyses. However, not many people exhibited a lot of interest in laboratory stock management prior to the 1990s. The NCCLS did not release document GP6-A, “Inventory Control Systems for Laboratory Supplies; Approved Guidelines,” until 1994. This document provides guidelines for inventory control systems to guarantee the availability of reagents and supplies in the laboratory [14].

Similarly, the Joint Commission International (JCI) accredits, according to its own standards, health organizations in more than 100 countries [15]. In this context, it has developed standards and guidelines for hospital laboratories [12].

The first national landmark that addresses quality in medical analysis laboratories was “Le Guide de Bonne Execution des Analyses Médicales” (or GBEA) in France. The initial decree (GBEA I), issued in Paris on November 2, 1994, dealt with the guidelines that medical laboratories were required to apply.

In contrast to American standards, the GBEA was designed to promote a work methodology which would lead to precise and accurate results rather than requiring a specific procedure to carry out an analysis. This methodology included all steps in the collection, processing, and delivery of a biological sample [16]. GBEA I required that procedures and analytical approaches be written [17]. The latter, related to quality control, was a key component of the quality assurance system in medical laboratories [16].

The second (GBEA II) constitutes a revision of GBEA I dated November 26, 1999 and released in the official journal on December 11, 1999 [18]. And it is annexed by a “Guide to the Good Use of Computers, GBUI” [17]. The GBEA contains texts targeting health in general, and particularly quality in medical biology in France. Moreover, it strongly empowers biologists, especially in the activities of the 2 phases, pre-analytical and post-analytical [18].

Then, ISO, whose technical committee 212 (ISO/TC 212) is organized by the NCCLS [10], established in 1999 the ISO 17025 standards “General requirements concerning the competence of laboratories for “calibration and testing”” [19]. These are a revision of “General requirements for the competence of calibration and testing laboratories” [20] and define the skills required to perform, using standardized or not, tests and/or calibrations, as well as the collection of samples in all laboratories. However, these standards do not rate the degree of proximity of laboratories to legal and safety recommendations [19].

On the other hand, in 2002, the NCCLS published “GP2-A4 Clinical Laboratory Technical Procedure Manuals; Approved Guideline – Fourth Edition,” which offers instructions on the development, review, approval, management, and use of policies, processes, and procedures for laboratory testing [14].

Then, in February 2003, the first edition of ISO 15189:2003, “Laboratories for Medical Analysis-Specific Requirements Concerning Quality and Competence,” was released, and rectified in July 2003 [21]. These standards are the first international requirements directed particularly to medical laboratories, and they include all of their application domains, unlike ISO 17025 standards, which strictly control the technical procedures for each analysis [1, 22]. The ISO/TR 22869:2005 Edition 1 recommendations were released in February 2005 to assist laboratories in implementing the 15189:2003 standards in European countries [23].

As a result, the GBEA was modified in France in 2004. “Metrology, IT, transport of biological specimens, molecular biology, and immunohematology” were the key areas affected by the changes [24]. Despite all of this development, an adequate level of “quality assurance system” cannot yet be achieved with the GBEA implementation alone [18]. In fact, the GBEA provides a regulatory text for a quality assurance system that, unlike the ISO 15189 standard, does not request a quality assurance manual and does not cover a number of concerns, such as personnel responsibility, contract review, subcontracted laboratories, continuous improvement, management review, etc. [24].

The GBEA assists hospital laboratories in organizing themselves during the accreditation process held by the National Agency for Accreditation and Evaluation in Health (Anaes) [18].

The safety control in laboratory procedures is also recognized in the US at the same time. The NCCLS published in 2004 the “HS1-A2 A Quality Management System Model for Health Care; Approved Guideline-Second Edition”, the “GP17-A2 Clinical Laboratory Safety; Approved Guideline-Second Edition” for the implementation of a safety program adaptable to any laboratory, and the “GP26-A3 Application of a Quality Management System Model for Laboratory Services; Approved Guideline-Third Edition”, which describes the path of activities in a clinical laboratory and provides the information needed to improve its processes and to meet government requirements and accreditation [14].

The ISO 17025:1999 standards were updated at the same time in May 2005 [25], and were corrected in 2006 [26]. The ISO/IEC 17025 standard, issued in 1999 as recommendations, was changed to requirements in 2005 [27]. Since the general structure of ISO 17025 and ISO 9001 are extremely similar, and since its 2005 version was developed to be compatible with the 2000 edition of the ISO 9000 series standards, laboratories that comply with it often work in accordance with those principles [28, 29]. Then, the ISO 15189:2007 standards, “Medical laboratories – Particular requirements for quality and competence” edition 2, were published in April 2007, and they were revised in September 2007 [30].

In April 2007 and September 2007, respectively, the ISO 15189:2007 standards, “Medical laboratories – Particular requirements for quality and competence” edition 2, were released and amended. “This International Standard, which is based on ISO/IEC 17025 and ISO 9001, specifies the competence and quality standards particular to laboratories doing medical biological analysis. It is recognized that a country may have its own particular regulations or requirements that may apply to all or a part of the profession and their related activities and obligations” [31]. The fourth edition of the “Quality Management System: A Model for Laboratory Services,” also known as the GP26-A4, was released in 2011 by the NCCLS, now known as the Clinical and Laboratory Standards Institute [10]. The two prior directives, HS01-A2 and GP26-A3, are combined in this iteration [32]. The ISO 15189 standard and the CLSI guidelines both focus on the quality checks that must be made throughout the full course of a laboratory’s work (in all three pre-analytical, analytical, and post-analytical phases) in order to produce a quality product or service [33].

Comparatively to the ISO 15189 standard, which essentially lists requirements without any more explanation, the CLSI GP 26 recommendations document provides significantly more detail on the integration of quality management in the various laboratory tasks. This document also discusses the laboratory’s sub-disciplines and offers advice customized to each. For this, these directives are internationally used for the implementation of the requirements of the ISO 15189 standard [12].

A model based on 12 key components of the quality system (QSE) – organization, customer orientation, premises and security, personnel, supply and inventory management, equipment, process management, documents and records, information management, management of nonconforming events (NCE) or problems, evaluations, and continuous improvement – provides a framework for the management of the laboratory work trajectory. This revised directive GP26-A4 was published in 2011 which describes an embedded system of QSEs and workflow paths. This edition has been reorganized to emphasize the importance of leadership responsibilities, reducing errors, and improving the efficacy and efficiency of activities in a laboratory. They function as building blocks to support the workflow path and various laboratory disciplines [32].

In September 2011, the JCI confirmed its concept’s alignment with key elements of the CLSI’s quality system (QSE) and/or the majority of the specific requirements of ISO 15189’s 2007 version, as well as the monitoring of the progress of their updates. JCI sets its standards beyond CLIA’s minimal requirements, like ISO [34]. The JCI Standards and Guidelines organize the activities of the different sectors of laboratory practice with the aim of providing quality standards for each sector or specialty [12].

Every five years, ISO standards are reviewed and updated. The third edition of the ISO 15189 standard for “Medical Laboratories-Requirements for quality and competence” was updated in 2012 and released in November of that same year [35].

As part of the several changes made to its 2007 edition, the standard’s 2012 revision specifies the process approach, tackles risk management, and uniformizes the management of laboratory information [36]. In addition to consistently monitoring patient and doctor satisfaction and loyalty, it defines criteria for documentation, enforces standards for automation guides, and requires the construction of a welcoming program for new hires [37].

Therefore, as of January 1, 2017, the third version of the JCI standards for the accreditation of hospital laboratories became operational. It contains sections on patient-centered standards and standards for managing healthcare organizations, which cover governance, leadership, and direction, information management, staff credentials and education, premises, and security, as well as the method for quality control process that takes into consideration the common areas and the specific one, like the quality control of specialties, laboratory examinations by specialties, as well as blood bank and transfusion [38].

In November 2017 [27], a new version of the ISO/IEC 17025:2005/Cor1:2006 standards were released, with a better focus on management, risk assessment, and prevention, in addition to process approach, system claims related to management system, decision-making rules, impartiality, competence, and consistency of laboratory operations. These criteria are appropriate for all institutions conducting laboratory activities [28]. This new version comprises two annexes, one detailing the potential for integrating an ISO 9001 management system and the other covering metrological traceability [39].

The laboratories targeted by this version are those that carry out calibrations, tests, and sampling; those that serve as first, second, or third parties; those that perform inspection operations or help with the certification process; those that have permanent facilities or mobile systems; and those that are part of an organization [17].

The patient and the prescribing doctor are not referenced in the ISO/IEC 17025 standard, despite the fact that technical requirements for quality management systems within it are comparable to those in ISO/IEC 15189 [29].

Then, a 4th edition of the JCI standards for the accreditation of hospital laboratories was published on January 1, 2022 [40].

Moreover, following revisions proposed and issued on February 4 2019, a new CLIA rule was published on July 11, 2022 in the Federal Register journal (87 FR 41194) entitled “Clinical Laboratory Improvement Amendments of 1988 (CLIA) Proficiency Testing Regulations Related to Analytes and Acceptable Performance”. This new rule was partially implemented in August 10, 2022 and will be applied from July 11, 2024 for the rest of the modifications [11].

After 10 years, the ISO 15189:2012 standard has been revised and finally “ISO 15189:2022 Medical laboratories – Requirements for quality and competence” edition 4 was published in December 2022. The latter is applicable to medical biology laboratories and to other medical laboratories such as pathological anatomy and cytology structures [41].

Several ISO standards dealing with requirements that could be added to the scope of medical laboratories’ work have also been developed. For example, ISO 13485:2016, “Medical devices – Quality management systems – Requirements for regulatory purposes” [42]; standard NF EN ISO 10012, “Measurement management system – Requirements for measurement processes and equipment” which addresses “the management of analytical instruments and processes” [22]; ISO 22870:2016 standard “Point-of-care testing (POCT) – Requirements for quality and competence” which deals with the conditions relating to offshore medical biology exams and which should be applied simultaneously with ISO 15189 standards by any healthcare institution offering “ambulatory care” [43].

The ISO 22870 standard’s initial edition was published in 2006, and updated in 2016 with minor revision that relates to the most recent version, ISO 15189: 2012.

The ISO 22870 standard applies when POCT are carried out “in a hospital or clinic and by a health organization providing ambulatory care” for “transcutaneous measurements, air analysis expired and *in vivo* monitoring of physiological parameters”. However, this guideline does not cover self-testing carried out by patients at home or in a clinic [43]. The operating room, emergencies, and intensive care are just a few of the hospital departments that are covered by the POCT measuring CPK, myoglobin and troponin [44] in addition to delocalized blood gases [45].

This quality management system supports the evaluation and approval of end-user proposals and protocols, the purchase and installation of equipment, “the maintenance of consumables and reagents; training, certification, and re-certification of users of POCT systems; quality control and quality assurance.” It also manages the risks for the patient and the health system [43].

Recently, the standards of this latest ISO 22870 standard have been incorporated into ISO 15189:2022 and the ISO 22870:2016 version has been canceled [41].

Currently standards are specific to laboratory activities such as the CAN/CSA-Z902 standard “Blood and blood products” drafted by the CSA Group for organizations “integrating a banking laboratory of blood, an autologous donation program, or a walking donor program,” are created specifically for specific industries or academic fields [1, 46]. Additionally, ISO 20776-1:2019 for the *in vitro* susceptibility of infectious pathogens and evaluation of the performance of devices for antibiograms – part 1: the broth microdilution reference method for assessing the *in vitro* susceptibility to antimicrobial agents of rapidly growing aerobic bacteria implicated in infectious diseases [47] or the H15 standard: Reference and Selected Procedures for the Quantitative Determination of Hemoglobin in Blood, Third Edition [48].

## Application of ISO 15189 standards

The ISO 15189 standard is now accepted as a fundamental reference for the accreditation of medical laboratories [41]. European Federation of Clinical Chemistry and Laboratory Medicine (EFLM) and International Federation of Clinical Chemistry and Laboratory Medicine (IFCC) admit that ISO 15189 is exactly representing standard for laboratories “EFLM and IFCC recognizes that ISO 15189 is precisely describing standard for labs*.*” [3].

Countries that opt for ISO 15189 to verify laboratory proficiency adopt a standardized procedure [49]. Additionally, since it might be optional, the government or another authority might advise adhering to the standard [1]. However, in countries where accreditation is not required, laboratories can seek accreditation from a foreign body [49].

For the accreditation of medical laboratories, only 59 % of accreditation bodies in European nations adopt the ISO 15189 standard as a reference [50, 51]. Whereas in the United States, before claiming accreditation to the ISO 15189 standard with CAP 15189, the laboratory must first be accredited in the CAP LAP which is based on the CLIA law, required in the United States. Indeed, ISO 15189 does not respond to CLIA claims and cannot replace CLIA certification [6]; ISO 15189 must retain a certain level of generality in order to be able to be adapted to several countries with different standards of practice framed mainly by the availability of resources [12].

However, laboratories that have been accredited in the CAP LAP can easily meet many of the technical requirements of the ISO 15189 standard, which is not the case for the more difficult to agree management requirements. However, since the implementation of management requirements undoubtedly has an effective role in improving the services provided [6], the ISO 15189 standard represents a universal spirit for laboratories in the United States which makes them potentially more competitive [33]. Thus, if in other countries such as the European Union, ISO standards have the ability to model governments, this is not the case in the United States [33] where the health system is based mostly on private health insurance providers [52]. For this, the CAP accredits more laboratories from other countries where ISO accreditation is the expectation of the paying government [33].

Indeed, CAP “accredits more than 8,000 laboratories in all its accreditation programs, including approximately 437 international laboratories in more than 50 countries” [6].

## Conclusions

The study of the standards related to medical laboratory and their evolution from the 1960s showed that standardization is now a crucial step and predict the orientations that this theme will take in the years to come.

The ISO 15189 standard represents the latest and most specific international standards for medical laboratories of all types. This standard currently constitutes a basic support for the majority of standards relating to the same field.

The outcomes of using such an approach show significant advantages for both laboratories and patients. Since the majority of therapeutic procedures are based on information provided by the medical laboratory, the effects on healthcare expenditures are undeniable.
